# Low Temperature Magnetic Transition of BiFeO_3_ Ceramics Sintered by Electric Field-Assisted Methods: Flash and Spark Plasma Sintering

**DOI:** 10.3390/ma16010189

**Published:** 2022-12-25

**Authors:** Alejandro Fernando Manchón-Gordón, Antonio Perejón, Eva Gil-González, Maciej Kowalczyk, Pedro E. Sánchez-Jiménez, Luis A. Pérez-Maqueda

**Affiliations:** 1 Instituto de Ciencia de Materiales de Sevilla, CSIC-Universidad de Sevilla, C. Américo Vespucio 49, 41092 Sevilla, Spain; 2 Departament de Química Inorgánica, Facultad de Química, Universidad de Sevilla, 41012 Sevilla, Spain; 3 Departament de Ingeniería Química, Universidad de Sevilla, Escuela Politécnica Superior, 41011 Sevilla, Spain; 4 Faculty of Materials Science and Engineering, Warsaw University of Technology, 141 Wołoska st., 02-507 Warsaw, Poland

**Keywords:** flash sintering, spark plasma sintering, bismuth ferrite, magnetic properties, mechanosynthesis

## Abstract

Low temperature magnetic properties of BiFeO_3_ powders sintered by flash and spark plasma sintering were studied. An anomaly observed in the magnetic measurements at 250 K proves the clear existence of a phase transition. This transformation, which becomes less well-defined as the grain sizes are reduced to nanometer scale, was described with regard to a magneto-elastic coupling. Furthermore, the samples exhibited enhanced ferromagnetic properties as compared with those of a pellet prepared by the conventional solid-state technique, with both a higher coercivity field and remnant magnetization, reaching a maximum value of 1.17 kOe and 8.5 10^−3^ emu/g, respectively, for the specimen sintered by flash sintering, which possesses the smallest grains. The specimens also show more significant exchange bias, from 22 to 177 Oe for the specimen prepared by the solid-state method and flash sintering technique, respectively. The observed increase in this parameter is explained in terms of a stronger exchange interaction between ferromagnetic and antiferromagnetic grains in the case of the pellet sintered by flash sintering.

## 1. Introduction

Multiferroic ceramic bismuth iron oxide (BiFeO_3_) has received considerable attention in the research community due to its unique properties of co-existence of ferroelectricity and ferromagnetism [1,2]. BiFeO_3_, in its bulk form, is a ferroelectric ceramic with a theoretical saturated polarization of 90 μC/cm^2^ and a relatively high Curie temperature $T_{C}\sim$1100 K [3]. At the same time, BiFeO_3_ exhibits an antiferromagnetic behavior related to the exchange interaction between Fe^+3^ ions up to the Néel temperature at approximately $T_{N}\sim$643 K [3]. However, bulk BiFeO_3_ suffers from high leakage current [4,5,6,7,8,9] and, generally, presents a non-homogeneous magnetic structure and a quadratic ferromagnetoelectric behavior, resulting in poor ferroelectric behavior and cancelling macroscopic magnetization [10,11].

To solve the problem of the poor magnetization of BiFeO_3_, different approaches have been addressed, such as the compositional substitution [12,13,14]. Moreover, the preparation of nanoscaled BiFeO_3_ samples has been revealed as an effective method for the enhancement of its magnetic properties [15,16,17,18]. Nevertheless, reported BiFeO_3_ nanostructures were prepared by the conventional die-pressing method, yielding porous materials with poor electrical properties, which unavoidably restricts their applications [1]. In this sense, Field-Assisted Sintering Techniques (FAST) have been shown to be useful techniques to sinter nanostructured ceramics, as they yield dense materials while minimizing grain growth [19,20]. Among FAST, Spark Plasma Sintering (SPS) [21] and Flash Sintering (FS) [22] techniques can be highlighted because it has been shown that nanostructured BiFeO_3_ can be prepared by both methods [15,23,24,25]. However, the magnetic behavior of this compound prepared by FS has not been reported.

The analysis of the phase transition behavior in BiFeO_3_ is commonly focused on a high-temperature regime and the nature of phase transitions below 300 K remain unclear. In this sense, magnetic measurements on single crystals, powders or nanostructured BiFeO_3_ have exposed different magnetic transitions within this temperature range [26,27,28]. Moreover, different experimental techniques, such as calorimetry, dielectric or mechanical measurements, as well as Raman spectroscopy have reported possible phase transitions close to 25, 38, 55, 140, 150, 178, 200 and 230-260 K [29,30,31], assigned to different phenomena, such as magnetic but glassy transitions (38–50 K), or magnetoelastic transition around 200–220 K. Therefore, it is of the most interest to perform more studies on the low-temperature regime in order to clarify the nature of the observed transitions. The present work is focused on studying the influence of the sintering process on the low-temperature magnetic behavior of bulk BiFeO_3_ sintered by two different FAST methodologies: flash sintering and spark plasma sintering. The obtained results were compared with those of a BiFeO_3_ specimen prepared by solid-state reaction.

## 2. Materials and Methods

BiFeO_3_ nanopowders were prepared by milling Fe_2_O_3_ (Sigma Aldrich, Darmstadt, Germany; <5 μm, ≥99% purity) and Bi_2_O_3_ (Sigma Aldrich, Darmstadt, Germany; ≥99.9% purity) commercial powder oxides using a high-energy planetary Fritsch Pulverisette 7 (Fritsch GmbH, Idar-Oberstein, Germany). A detailed information on the procedure can be found elsewhere [32]. The mechano-synthesized powders were subsequently sintered by two techniques: flash-sintering and spark plasma sintering. The flash-sintering experiments were carried out using the standard procedure [24]. The sample was flashed at 100 V cm^−1^ and 20 mA mm^−2^ for 15 seconds, with the flash event occurring at 773 K. On the other hand, the SPS experiment was carried out in a commercial SPS Model 515S (SPS Dr Sinter Inc., Japan) under vacuum using a pressure of 75 MPa at 898 K for 10 min. In reference [10], more detailed information about the sintering process by SPS can be found. For comparison purposes, a bulk BiFeO_3_ pellet was prepared by conventional solid-state reaction using the same commercial powders mixed in an agate mortar for ~10 min and uniaxially pressed to prepare a cylindrical pellet. The specimen was fired at 1123 K for 0.5 h using a heating rate of 10 K min^−1^ in an alumina boat placed on powder of the same composition. 

The structure of the obtained pellets was studied by X-ray diffraction, XRD, at room temperature, using Cu-Kα radiation in a Rigaku MiniFlex diffractometer (Tokyo, Japan). Phase transition temperatures were analyzed by differential scanning calorimetry using a simultaneous TG/DSC (Q650 SDT; TA Instruments, New Castle, DE 19720, USA) under a nitrogen flow and 10 K min^−1^ heating rate. Microstructural characterization was carried out by scanning electron microscopy in a Hitachi S-4800 microscope (Tokyo, Japan).

Magnetic characterization of the pellets was carried out using the standard vibrating sample magnetometer option of a Physical Properties Measurement System, PPMS, (Quantum Design, San Diego, CA, USA) applying an external magnetic field of 100 Oe, a heating/cooling rate of ±1 K/min in zero-field cooling (FC), field heating (FH) and field cooling modes.

The in situ evolution of the crystallographic structure of the samples with temperature from room temperature to 180 K (on cooling and heating) and with a heating rate of 10 K/min was measured in a Bruker D8C diffractometer (Bruker, Billerica, MA, USA) with Cu-Kα radiation. Each pattern was collected at the selected temperature (measured time less than 5 min). Phase identification and Le Bail refinements were performed by DIFFRAC.EVA (version 6, Bruker, Billerica, MA, USA) and DIFFRAC.TOPAS (Version 6; Bruker, Billerica, MA, USA) software, respectively.

## 3. Results and Discussion

**Figure 1:**
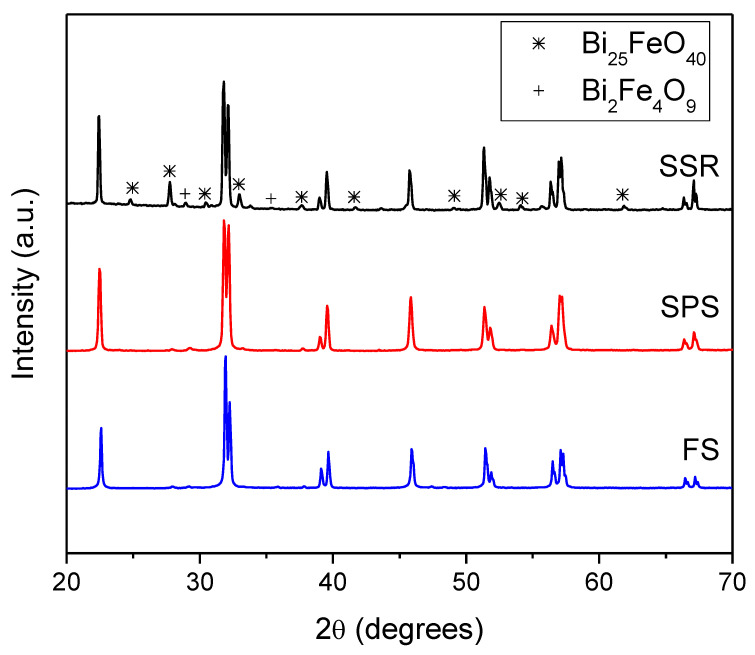
XRD patterns, taken at room temperature, of the BiFeO_3_ specimens prepared by the solid-state reaction method (SSR) and by mechanosynthesis and subsequently densified by SPS and FS.

Figure 1 shows XRD patterns, taken at room temperature, of the three studied sintered specimens. All the diffraction peaks of BiFeO_3_ specimens sintered by non-conventional methods can be indexed as a rhombohedral perovskite structure with an R3c space group, which indicates the retention of pure BiFeO_3_ after the sintering process. By contrast, the conventionally sintered sample partially decomposed into secondary Bi_25_FeO_40_ and Bi_2_Fe_4_O_9_ phases. In fact, these secondary phases were often observed in this compound, as BiFeO_3_ is metastable and decomposes at relatively low temperatures [33,34].

**Figure 2:**
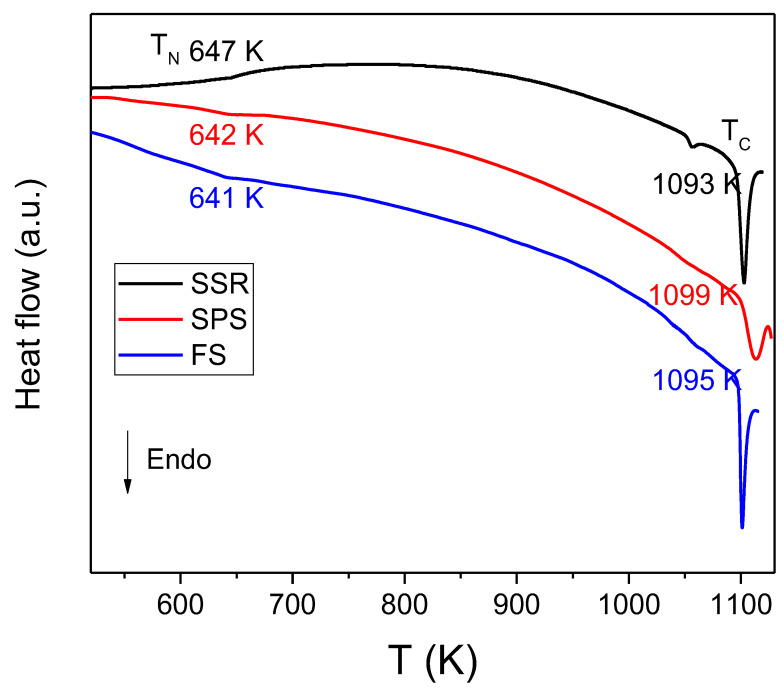
Differential scanning calorimetry (DSC) curves of BiFeO_3_ prepared by SSR and by mechanosynthesis and subsequently densified by SPS or FS.

The multiferroic character and homogeneity of the obtained pellets were studied by DSC in a non-isothermal regime, since phase transition temperatures vary with the existence of impurities [34]. Figure 2 shows the DSC scans taken at 10 K min^−1^ on heating. All samples exhibited a weak transition at around 643 K. Considering the data reported in the literature, this peak corresponds to the antiferromagnetic–paramagnetic transition of the samples, i.e. the Néel temperature. A much more intense endothermic peak appeared around 1093 K. It is associated with the ferroelectric-paraelectric transition and determines the Curie temperature. The temperature at which both transitions were observed are in very good agreement with those reported in the literature for high-quality BiFeO_3_ [4,32,35]. Additionally, for the sample prepared by SSR, a third endothermic peak can be clearly observed at approximately 1057 K. This peak has been related to the existence of impurities, in agreement with the XRD data.

**Figure 3:**
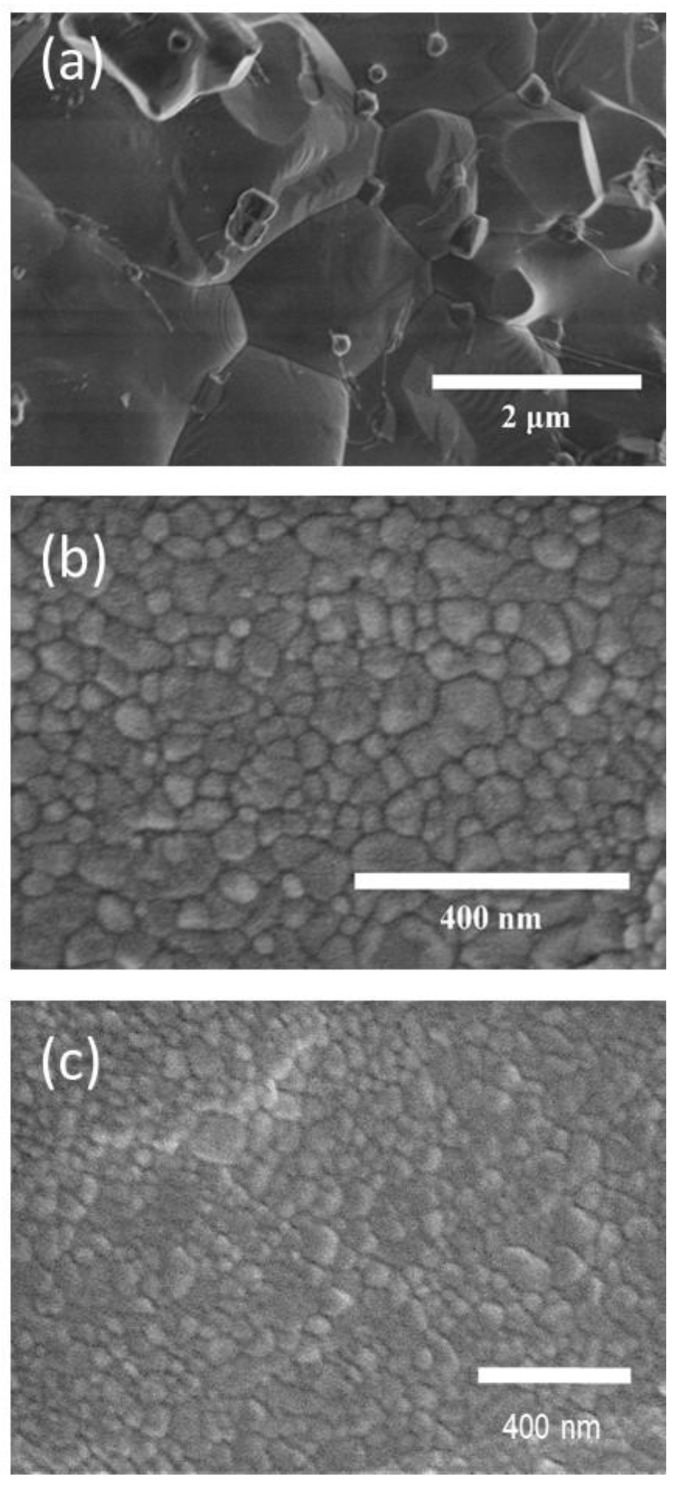
Scanning electron microscopy micrographs of pellets prepared by (**a**) SSR, and mechanosynthesis followed by sintering using (**b**) SPS and (**c**) FS.

SEM micrographs of the sintered pellets are presented in Figure 3. In the case of the pellet sintered conventionally, the micrograph shows large grains, typically of 2.5–10 μm. By contrast, the pellet sintered by SPS exhibits a microstructure with a grain size of 100 ± 20 nm. Finally, the microstructure of the pellet sintered by FS corresponds to a well-sintered material with smaller grains of an average size of 40 ± 12 nm.

**Figure 4:**
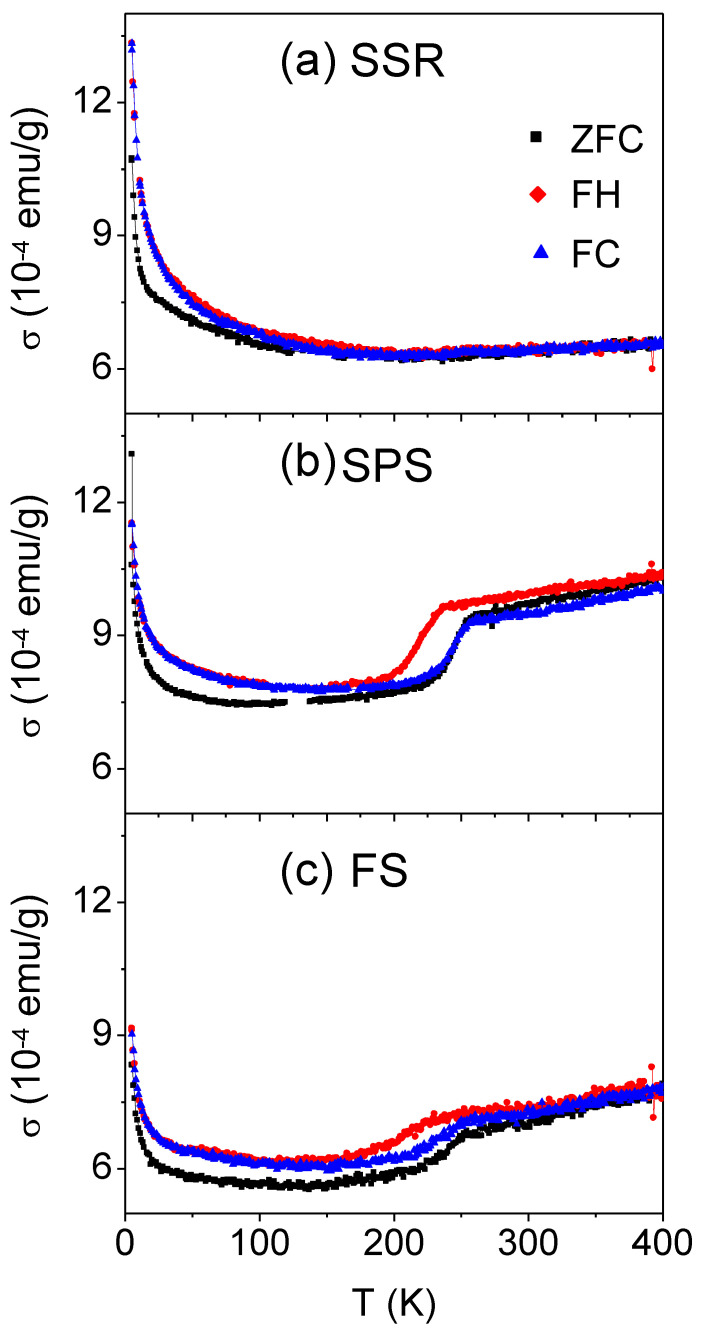
Temperature dependence of the magnetization of the specimens prepared by (**a**) SSR, and mechanosynthesis and sintered by (**b**) SPS and (**c**) FS, depicting ZFC, FH, FC curves, with an external applied magnetic field of 100 Oe.

The magnetic behavior of the BiFeO_3_ samples was analyzed under an externally applied field of 100 Oe through zero-field cooling (ZFC) and field cooling (FC) curves. The field heating (FH) curve is also shown (see Figure 4). For the SSR sample, a plateau-like shape can be observed in the whole temperature range, although there are some anomalies. The irreversibility of magnetization was evidenced at temperatures below 100 K, i.e., the ZFC and FC curves split below this temperature. Such splitting phenomena is commonly attributed to ferromagnetic and antiferromagnetic interfaces [26], and it has been observed in other BiFeO_3_-related compounds [15,27,36,37]. As can be seen, this phenomenon is more remarkable for the BiFeO_3_ nanoceramic samples sintered by SPS and FS as compared to the sample prepared by SSR. This fact is related to the increase in the ferromagnetic-antiferromagnetic interfaces due to the decrease in the grain size of the specimens prepared by FAST methodologies [26]. These interfaces are also the reason for the existence of the exchange bias (EB) effect (this effect will be discussed below). Furthermore, both ZFC and FC curves depict a significant increase at temperatures below ~20 K due to the weak ferromagnetism of BiFeO_3_ at these temperatures [38,39]. Although this magnetization enhancement can be observed at low temperatures for the three studied specimens, it is higher in the case of the sample prepared by the conventional method, probably due to the magnetic contribution of the parasitic phases [40].

Interestingly, in addition to the features discussed above, which are common to all specimens, the magnetization curves of the sample densified by SPS present an anomaly of about 250 K, which suggests the occurrence of a phase transformation. The thermal hysteresis between FC and FH curves, typically observed in first-order type transitions [41], might indicate the magnetoelastic nature of this transition. In fact, the temperature range assigned to magnetoelastic transition in previous works (although not by magnetic measurements) [29,30,31] is in good agreement with the anomaly observed at approximately 250 K for the sample sintered by SPS. The magnetization curves of the specimen densified by FS are quite similar to those of the sample densified by SPS. Nevertheless, the possible magnetoelastic transition is weaker. This could be related with the decrease in the grain size. In fact, it has been reported that below a critical grain size the magnetoelastic transition can be suppressed in different systems [42,43,44].

For the purpose of exploring the nature of the magnetic transition found at around 250 K and to obtain a better understanding of the phase evolution for each sample, temperature-dependent X-ray diffraction patterns were registered. Figure S1 (Supplementary Materials) depicts the XRD patterns of the sample sintered by SPS on cooling and heating, and registered in situ between 180–300 K. In the entire studied temperature range, no modification of the crystal structure of BiFeO_3_ occurred; only the expected shift of the peaks to lower angles as the temperature was lowered from 300 to 180 K can be observed.

**Figure 5:**
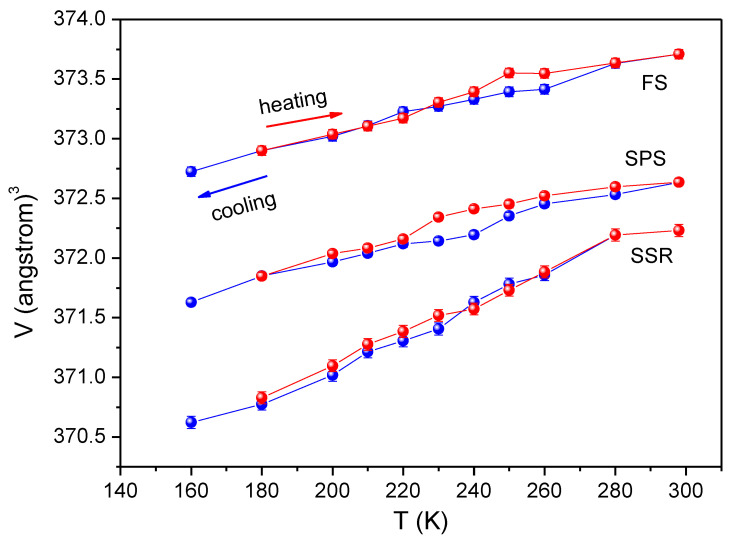
Cell volume of BiFeO_3_ as a function of temperature for all the studied samples.

XRD patterns were analyzed by Le Bail refinement (goodness of fit, GOF ≤ 1.6). Figure 5 depicts the evolution of cell volume of the BiFeO_3_ phase with temperature, where a significant deviation from the trend is detected for the sample sintered by SPS. This deviation, which is not distinguished in the case of the SSR sample and is less defined in the case of the FS-ed sample, is accompanied by certain thermal hysteresis between both heating and cooling. These facts support that the transformation corresponds to a first-order phase transition inferred from the behavior of the magnetization curves (see Figure 4). In fact, there is a modification of the volume of the cell without a change of the crystal structure. The obtained results allow us to determine the magnetoelastic nature of the observed transition, which has been previously attributed to the existence of impurities [45] or, more recently, described as a magnetic but glassy transition [37].

**Figure 6:**
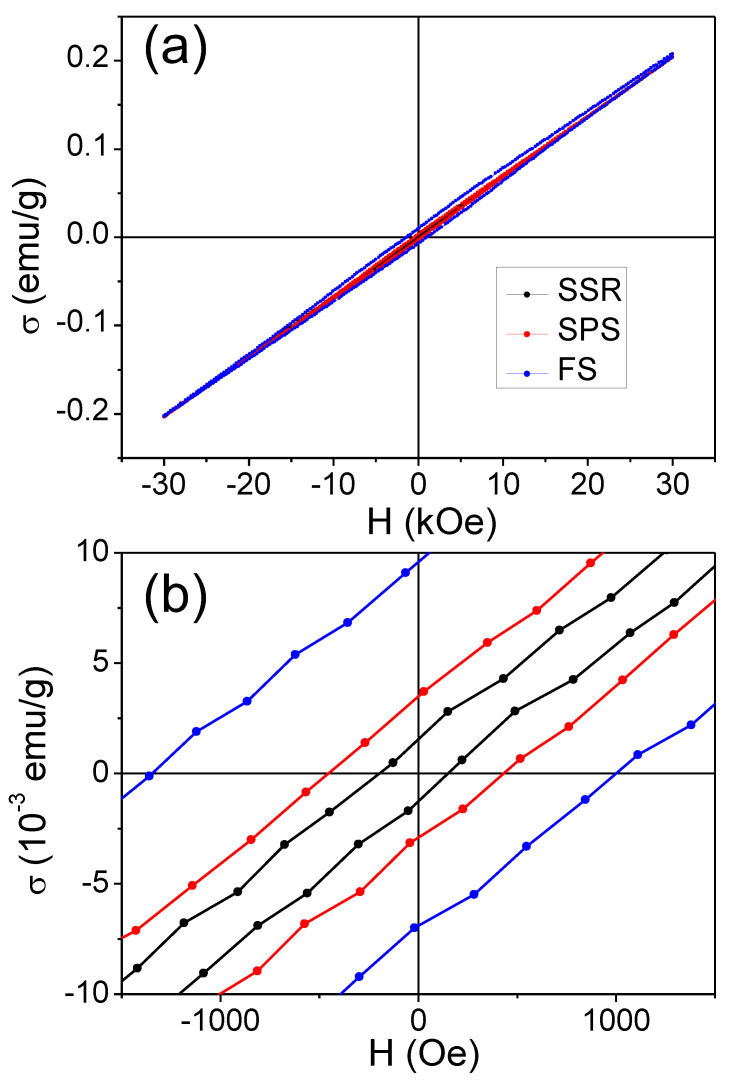
(**a**) Magnetic hysteresis loops of the studied specimens taken at 300 K. (**b**) Low field region of the hysteresis loops.

Once the magnetic behavior at low temperature of the studied specimens has been analyzed, Figure 6 shows the magnetic hysteresis loops at 300 K. As expected, the specimens show an almost linear field dependence of magnetization due to the G-type antiferromagnetic behavior of BiFeO_3_, especially in the case of the sample prepared by SSR, implying that the magnetization (or remnant magnetization) is practically zero. This behavior of the hysteresis loops agrees with the expected antiferromagnetic nature of the studied compound. The reduction in grain size (see micrographs in Figure 3) leads to the appearance of some hysteresis in the case of the samples sintered by FAST, with a major effect in the case of the specimen sintered by FS. The improvement of magnetic properties in nanostructured BiFeO_3_ is currently under discussion, and three principal factors are under consideration: a partial compensation of antiferromagnetic sublattices at the surface, an increase in the spin canting angle of Fe-O-Fe bonds introduced by strain and an annihilation of the spiral spin structure [36,46,47]. Even though there are no important discrepancies in the maximum magnetization at the range of magnetic fields studied, differences in remnant magnetization $\sigma_{r}$ and coercivity $H_{C}$ can be highlighted. Indeed, $\sigma_{r}$ reached the highest value at 8.5 10^−3^ emu/g for the specimen sintered by FS, which possessed the smallest grains.

The exchange anisotropy existing at the interface between ferromagnetic and antiferromagnetic grains can originate exchange bias (EB) phenomena. In the case of BiFeO_3_, this effect can appear at the interface as a result of the interaction between the ferromagnetic grains with a size smaller than 62 nm, and antiferromagnetic grains. The value of EB, $H_{EB}$, can be determined as $H_{EB}\;$=$\;\left(H_{C+}+H_{C-}\right)/2$, where $H_{C+}$ and $H_{C-}$ are the positive and negative fields when magnetization is zero, respectively [48]. The obtained values are collected in Table 1. A relatively low EB effect is observed for the BiFeO_3_ sample prepared by SSR, whereas it increases for the specimens sintered by FAST techniques, i.e., with the decrease in particle size. Thus, EB is ~−110 Oe for the specimen sintered by SPS and~−177 Oe for the FS specimen. In this latter sample, the exchange interaction between ferromagnetic and antiferromagnetic grains is responsible for the larger EB effect. Once $H_{EB}$ is known, $H_{C}$ can be correctly determined, the values of which have been also collected in Table 1. It can be observed that the increase in coercivity is caused by a drop of the particle size. This reduction provokes the generation of a single magnetic domain of the grains. In this way, the mechanism that generate the magnetic behavior changes from domain wall motion to magnetization rotation [36]. Moreover, a more important role of surface anisotropy effects could be expected with the decrease in the particle size [49].

For comparison purposes, Table 1 also includes data from the literature of BiFeO_3_ samples sintered by SPS (data for samples sintered by FS have not been found) [15,23,50]. It can be clearly observed that the presented parameters are comparable to those obtained in this work for the SPS sample. It is worth noting that the BiFeO_3_ specimen prepared by FS displays an enhanced ferromagnetic character compared to those prepared by SPS. Generally, it is assumed that the increase in the magnetic parameters is due to the suppressed magnetic spin structure when the grain size is below ~62 nm [54]. On the other hand, the magnetism of BiFeO_3_ can be tailored by structural modifications by the addition of different types of substituents, as can be seen in Table 1.

## 4. Conclusions

Pellets of dense and phase-pure BiFeO_3_ obtained by mechanosynthesis and sintered by flash sintering, FS and spark plasma sintering, SPS, were characterized by magnetization measurements. The results were compared with those obtained for a sample prepared by a conventional solid-state reaction. It is worth emphasizing that the magnetic behavior of a BiFeO_3_ specimen sintered by FS has not been previously described.

Low-temperature magnetic behavior indicates the co-existence of superparamagnetic relaxation phenomena, which imply the splitting of magnetization curves at low temperatures introduced by strong interparticle interactions (<100 K). Interestingly, zero field-cooled, field-heated and field-cooled magnetization curves revealed a phase transition at around 250 K in specimens densified by field-assisted sintering techniques, which is particularly remarkable in the sample prepared by spark plasma sintering. The magnetoelastic nature of this transition, thermal hysteresis between both heating and cooling processes and modification of the volume without crystal structure variation, are supported by in situ XRD measurements.

## Figures and Tables

**Table 1:** Magnetic properties of BFO-based bulk ceramics sintered by electric field assisted-methods.

Composition	Technique	d	$\sigma_{r}$ (10^−3^ emu/g)	$H_{EB}$ (O_e_)	$H_{C}$ (O_e_)	Reference
BiFeO_3_	Solid-State Reaction	data	1.3	−22	177	This work
	Mechanosynthesis + SPS	~100 nm	3.5	−11	451	
	Mechanosynthesis + FS	~30 nm	8.5	−177	1173	
BiFeO_3_	Sol-gel + SPS	~110 nm	11	500 (5K)		[15]
BiFeO_3_	Sol-gel + SPS	1–3 μm	0.6		50	[50]
		1–3 μm	2.4		120	
BiFeO_3_	High-energy ball milling + SPS	<200 nm	5.7		600	[23]
BiTi_0.05_Fe_0.95_O_3_	Sol-gel + SPS	<100 nm	10		500	[51]
Bi_0.85_La_0.15_FeO_3_	High-energy ball cryo milling + SPS	24 nm	5.2		630	[52]
Bi_0.95_Nd_0.05_FeO_3_	Sol gel + SPS	<1 μm	10		685	[53]
Bi_0.90_Nd_0.10_FeO_3_			101		6721	
Bi_0.85_Nd_0.15_FeO_3_			181		9497	
Bi_0.95_Sm_0.05_FeO_3_			21		1954	
Bi_0.90_Sm_0.10_FeO_3_			133		9627	
Bi_0.85_Sm_0.15_FeO_3_			279		15117	

## Data Availability

Data will be available on request.

## Supplementary Materials

The following supporting information can be downloaded at: https://www.mdpi.com/article/10.3390/ma16010189/s1, Figure S1: XRD patterns as a function of temperature from 300 to 180 K for the sample prepared by SPS on cooling (upper panel) and heating (lower panel).
