# Supplementary material for: Low Temperature Magnetic Transition of BiFeO3 Ceramics Sintered by Electric Field-Assisted Methods: Flash and Spark Plasma Sintering
Source: Materials (Basel). 2022 Dec 25;16(1):189. doi: 10.3390/ma16010189 (PMC9821829; doi:10.3390/ma16010189)
Supplement: Supplementary file 1 [file materials-16-00189-s001.zip › materials-2112087-SI.pdf]

Supplementary

Low Temperature Magnetic Transition of $BiFeO_3$ Ceramics Sintered by Electric Field-Assisted Methods: Flash and Spark Plasma Sintering

Alejandro Fernando Manchón-Gordón *, Antonio Perejón *, Eva Gil-González, Maciej Kowalczyk, Pedro E. Sánchez-Jiménez and Luis A. Pérez-Maqueda

Figure S1 shows in situ XRD patterns of the $BiFeO_3$ sample sintered by SPS as a function of temperature (between 180 and 300 K) on cooling and heating at 10 K/min. All the studied samples exhibit the structure with R3c space group as a single phase in all the temperature studied range.

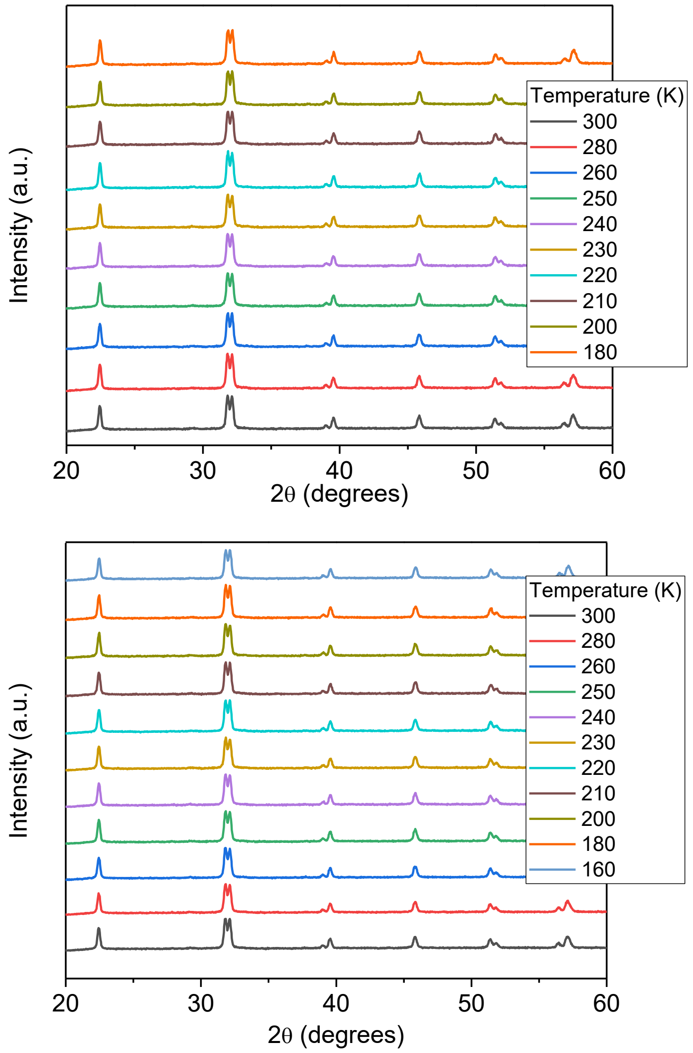

Figure S1. XRD patterns as a function of temperature from 300 to 180 K for the sample prepared by SPS on cooling (upper panel) and heating (lower panel).
